# Total arsenic and speciation analysis of saliva and urine samples from individuals living in a chronic arsenicosis area in China

**DOI:** 10.1186/s12199-017-0652-5

**Published:** 2017-05-11

**Authors:** Dapeng Wang, Yasuyo Shimoda, Sanxiang Wang, Zhenghui Wang, Jian Liu, Xing Liu, Huanyu Jin, Fenfang Gao, Jian Tong, Kenzo Yamanaka, Jie Zhang, Yan An

**Affiliations:** 1 Department of Toxicology, School of Public Health, Jiangsu Key Laboratory of Preventive and Translational Medicine for Geriatric Diseases, Medical College of Soochow University, Suzhou, Jiangsu 215123 People’s Republic of China; 2 Key Laboratory of Environmental Pollution Monitoring and Disease Control, Ministry of Education, Department of Toxicology, School of Public Health, Guizhou Medical University, Guiyang, Guizhou China; 3 Laboratory of Environmental Toxicology and Carcinogenesis, School of Pharmacy, Nihon University, Chiba, Japan; 4 Shanxi Institute for Prevention and Treatment of Endemic Disease, Linfen, Shanxi People’s Republic of China

**Keywords:** Arsenic speciation, Saliva, Urine, Drinking water, Biomarker

## Abstract

**Background:**

It is generally acknowledged that the determination of harmful chemical compounds excreted into saliva is useful for assessing their exposure levels. The aim of the present study was to compare the total arsenic and its species in saliva and urine samples collected from the people residing in an arsenic-contaminated area of China and to further verify the feasibility of using salivary arsenic as a new biomarker of arsenic exposure.

**Methods:**

Total arsenic and speciation analyses in urine and saliva samples among 70 residents exposed to arsenic from drinking water in Shanxi, China were carried out by high-performance liquid chromatography-inductively coupled plasma-mass spectrometry (HPLC-ICP/MS).

**Results:**

The result showed that, total arsenic concentration in saliva was relatively lower than in urine samples, but it existed a strong positive correlation with total urinary arsenic, drinking water arsenic and different skin lesions. For arsenic metabolism analyses, As^III^, As^V^, MMA, and DMA were detected in all of the urine samples with the dominating species of DMA (73.2%). Different with urinary arsenic species, most arsenic species in saliva were not methylated. The major species in saliva was iAs (As^III^ + As^V^, 76.18%), followed by DMA (13.08%) and MMA (9.13%). And the primary methylation index (PMI), second methylation index (SMI) and proportion of the four different species (As^III^, As^V^, MMA, and DMA) in saliva showed no significant positive relationship with that of in urine.

**Conclusions:**

These findings indicated saliva may be used as a useful tool for biological monitoring of total arsenic exposure in the crowd rather than an efficient tool for assessing arsenic metabolism in human body after exposed to arsenic.

## Background

Arsenic is a ubiquitous element in the earth’s crust [1] and is widely distributed in water, air, soil, and food in both inorganic and organic forms [2]. Inorganic arsenic (arsenate and/or arsenite) has long been recognized as human carcinogens by the International Agency for Research on Cancer (IARC) [3]. Long-term exposure to inorganic arsenic can cause numerous human health effects, including several types of cancers [3–5], cardiovascular disease, and diabetes [6, 7]. In addition, arsenicosis is a serious and widespread global public health problem [8] with more than 200 million people at risk of toxic arsenic exposure from ground water and food contamination [9]. Considerable progress has been made in recent years to address arsenic toxicity, including both genetic and epigenetic alteration [10, 11]. In spite of these efforts, the exact molecular and cellular mechanism involved in arsenic toxicity are rather unrevealed given that the complicated metabolism of arsenic in the human body, and no effective treatment for arsenicosis exists [3]. Hence, timely screening for arsenic exposure and accessing arsenic metabolism is particularly vital in preventing arsenic poisoning. Traditionally, samples for screening arsenic exposure mainly include blood, urine, hair, and nails [12, 13]. More recently, salivary analyses has became a useful tool for disease diagnosis because of its non-invasive collection method and easy storage [14]. Additionally, in the area of biological monitoring, previous studies on the use of saliva have focused on lead, cadmium, mercury, and herbicide concentrations in humans or animals [15–19]. For arsenic exposure, there have been limited and paradoxical studies that have detected arsenic concentration in saliva. Yuan et al., [20] first analyzed arsenic and its species in human saliva from an arsenic-contaminated area and found that salivary arsenic could be a potential biomarker of arsenic exposure. Subsequently, other studies carried out in India and Thailand had shown that total arsenic concentrations in saliva have an evident positive correlation with the total arsenic levels both in urine and drinking water [21, 22]. In a different study, Lew et al. [23] did not find any significant differences in the concentration or speciation of arsenic in saliva samples from children that were exposed to arsenic by playing in Chromated Copper Arsenate (CCA) treated wood playground compared to those that did not play in CCA-treated wood. Moreover, some researchers believed that the low concentration of arsenic and small variations made salivary arsenic unsuitable as a biomarker of arsenic exposure [24]. Hence, whether or not saliva can be used as an efficient tool for biological monitoring of arsenic exposure, especially for assessing arsenic species remains to be further verified. The aim of the present study, therefore, was to compare the total arsenic and its species in saliva and urine samples collected from the people residing in an arsenic-contaminated area of China and to further verify the feasibility of using salivary arsenic as a new biomarker of arsenic exposure.

## Methods

### Study population

The National Diagnosis Standard for Endemic Arsenism (WS/T211-2001) [25], was used by trained biomedical personnel from Shanxi Institute for Prevention and Treatment of Endemic Disease to identify and categorize cases of arsenicosis during the survey. According to our previous survey data [26, 27], we chose 42 families (total of 70 individuals) from 4 villages in an endemic arsenicosis area in Shanyin County, Shanxi Provence, China, as subjects, and then collected well water samples from all families, and urine and saliva samples from all individuals. This study was approved by the Ethics Committee of the School of Public Health, Medical College of Soochow University according to the recommendations of the World Medical Association Declaration of Helsinki Ethical Principles [28] for international health research. All participants provided informed consent before participating.

### Sample collection

Water samples were collected from all the families into polyethylene bottles rinsed beforehand with dilute nitric acid and then 5 ~ 6 drops of 30% nitric acid were added to maintain the pH value below two. Urine samples were collected into disposable plastic cups and then dispensed into 50 mL polyethylene centrifugal tubes cleaned with dilute nitric acid and kept on ice. The saliva samples were collected at least 1 h after any food consumption, before collection, participants were told to rinse their mouths at least 3 times to remove any food residue, then threw away some of the initial saliva, collected 2 mL of the saliva into centrifuge tubes and kept on ice. All samples were stored at −80 °C until analysis.

### Chemicals

Analytical grade reagents were used throughout. Arsenic standard solution for atomic absorption spectrometry (1000 μg/mL), the chemical standards of sodium arsenite (As^III^), sodium arsenate (As^V^), methylarsonic acid (MMA), arsenobetaine (AsBe) were purchased from Wako Pure Chemical Industries, Ltd. (Osaka, Japan). Dimethylarsinate (DMA) was obtained from Tri Chemical Laboratory (Yamanashi, Japan). Germanium standard solution (Kanto Chemical, Tokyo, Japan) was used as internal standard for HPLC-ICP/MS analysis. Nitric acid (HNO3, Wako Pure Chemical, Osaka, Japan) was used for sample treatment. Ammonium bicarbonate (NH4HCO3, Bio Ultra, Sigma-Aldrich, St. Louis, MO, USA) and methanol (for high performance liquid chromatography, Wako Pure Chemical, Osaka, Japan) was used for the mobile phase of HPLC. Tap water was purified through Milli-Q Plus (Millipore Japan, Tokyo, Japan). The certified reference material, NIES CRM No. 18 (human urine), from the National Institute for Environmental Studies (Ibaraki, Japan) was used to validate the analytical procedure.

### Sample preparation and total arsenic detection

For water samples, 1 mL of them was aspirated into polyethylene centrifugal tubes and then 5 mL of HCl and 5 mL of 2% thiourea-ascorbic acid mixture were added to produce the final volume, an Atomic Fluorescence Spectrometer (AFS-230, Beijing Kechuang Haiguang Instrument Corporation, China) was used for total arsenic detection.

The urine and saliva samples were diluted three-fold with 0.1 M nitric acid and centrifuged at 3000 rpm for 10 min. The supernatants were sonicated for 30 min, then filtered through a 0.45 μm polyvinylidene fluoride filter (Whatman 13 mm GD/X syringe filter; Whatman, Florham park, NL, USA) prior to total arsenic analysis. The method was validated by analysis of NIES CRM No.18. The total arsenic concentration of the reference material was detected to be 141.30 ± 3.80 μg/L (*n* = 5), which was within the range for the certified value of 137.00 ± 11.00 μg/L. After preparation, the acid-digested solution was diluted with ultra-pure water and introduced into an Agilent 7500a ICP-MS (Agilent Technologies, Santa Clara, CA, USA). The instrument settings were as follows: radio-frequency (RF) power, 1400 W; argon plasma gas flow, 15 L/min; and carrier gas (argon) flow, 1.10 L/min. A concentric type nebulizer, nickel skimmer, and sample cones were used, and detection mass was set to m/z of 75 (75As+) and 77 (40Ar37Cl+). The instrument limit of detection (LOD) of aqueous arsenic standard solution and the method limit of quantitation (LOQ) were calculated according to the definition stipulated by Japanese Industrial Standards (JIS) [29]. The LOD and LOQ were calculated as 0.20 and 1.10 μg/L, respectively.

### Determination of arsenic species in urine and saliva by HPLC-ICP-MS

After centrifugation, the supernatant were diluted five-and one-point-five-fold with ultra-pure water, respectively, and then filtered through a 0.45 μm polyvinylidene fluoride filter (Whatman 13 mm GD/X syringe filter). After preparation, an Agilent 1100 HPLC series (Agilent Technologies) with a Dionex IonPac AS22 column (250 × 4.0 mm i.d., Thermo Fisher Scientific, Waltham, MA, USA) and an Agilent 7500a ICP-MS were used to separate and detect the arsenic species. The HPLC separation conditions were as follows: Mobile phase, 20 mM NH4HCO3 (pH 10.0); Flow rate, 1.20 mL/min; Column temperature, 40 °C, and Injection volume, 50 μL. The LOD of As^III^, As^V^, MMA, DMA, and AsBe were calculated as 0.90, 0.50, 0.70, 0.70, and 0.70 μg/L, respectively. The AsBe and DMA concentrations in the CRM No.18 urine were 77.70 ± 2.70 μg/L and 39.00 ± 1.10 μg/L (*n* = 5), and the values were within the ranges for certified value of 69.00 ± 12.00 and 36.00 ± 9.00 μg/L, respectively.

### Creatinine (Cr) in urine

Urinary creatinine was measured by Microplate Reader (BioTek Instruments, Inc., USA) using a Metra Creatinine Assay Kit (Beijing Kinghawk Pharmaceutical CO., LTD, China). Concentrations of total arsenic and its species in urine were normalized by urinary creatinine concentrations.

### Statistical analysis

Data analysis was carried out using SPSS software (version 17.0, SPSS Inc., Chicago, IL, USA). The statistical significance for the different groups was determined using one-way analysis of variance (ANOVA) or Student’s *t*-test. Histogram and normal probability plot of the arsenic concentration in drinking water and saliva revealed that the distributions were normality, and in urine it was right skewed and deviated from normality, so the bivariate associations were analyzed by Pearson and/or Spearman’s rank correlation analysis. Any *p*-values less than 0.05 were considered statistically significant.

## Results and Discussion

### The characteristics of human subjects and distribution of total arsenic in drinking water

Arsenicosis has long be recognized as a major public health issue in the world and Shanyin County in Shanxi Province is one well known endemic area of severe arsenicosis in China [30, 31]. In the present study, 70 participants from 42 families in Shanyin County of Shanxi Province, China were recruited, and approximately half of them were male (37 males and 33 females). The average age of the participants was 48 years old with the range from 21 to 78. In addition, 42 well water samples were also collected from all families and analyzed the total arsenic by AFS-230, the highest and lowest concentrations of arsenic were determined to be 720.00 μg/L and 0.55 μg/L, respectively. The median value of arsenic in drinking water samples was 127.22 μg/L, and 66.67% of the arsenic levels exceeded the drinking water standard in China for arsenic (50 μg/L) [32], the results that were similar to our previous experimental results [26, 27] (Table 1).

**Table 1:** Distribution of total arsenic in drinking water, saliva and urine from individuals exposed to different levels of arsenic in drinking water in Shanyin County of Shanxi Province, China

Arsenic levels in drinking water (μg/L)	Skin lesions (n)	Arsenic concentration (μg/L)	Salivary arsenic (μg/L)	Urinary arsenic (μg/gCr)
	Absent/Present	Median (Min ~ Max) (n)	Median (95%CI) (n)	Median (95%CI) (n)
<50	17/4	11.15 (0.55 ~ 41.86) (14)	3.41^a^ (0.96–18.54) (21)	53.20^a^ (7.53–330.71) (21)
50–200	10/12	125.61 (73.25 ~ 199.92) (13)	11.20^b^ (2.78–44.03) (22)	123.34^b^ (32.40–435.93) (22)
>200	6/21	317.63 (204.16 ~ 720.00) (15)	22.91^c^ (9.56–62.11) (27)	167.70^c^ (103.95–723.10) (27)
Sum	33/37	127.22 (0.55 ~ 720.00) (42)	12.31 (1.86–46.62) (70)	124.93 (21.80–497.70) (70)

Values represent the median (95%CI). Statistical analysis using *t*-test. Median values within a column not sharing a common superscript letter (a, b, c) were significantly different, *P* < 0.05

### Total Arsenic concentration in saliva and urine of individuals

The present study used ICP-MS to detect total arsenic in the saliva and urine of 70 individuals. The detection results showed that the median value of salivary arsenic and urinary arsenic was 12.31 μg/L and 124.93 μg/gCr, respectively (Table 1). We divided the water arsenic concentrations into three groups: <50 (μg/L), 50 ~ 200 (μg/L) and >200 (μg/L). Comparison of the arsenic concentrations in urine and saliva among the three groups showed that the urinary arsenic level increased gradually with an increase of the arsenic concentration in drinking water; the median values of urinary arsenic in the 3 groups were 53.20, 123.34 and 167.70 μg/gCr, respectively. There was a significant difference among the 3 groups, *P* < 0.05 (Table 1). Urinary arsenic is currently accepted as the biomarker of arsenic exposure. Our results also confirmed that urinary arsenic can reflect the level of exposure to arsenic, which was consistent with the results of urinary arsenic in children and adults exposed to arsenic in drinking water in Inner Mongolia as reported by Sun et al. [33]. Our study analyzed the total arsenic in human saliva of three groups, and found that, similar to urinary arsenic, with an increase in the arsenic concentrations in drinking water, the salivary arsenic level increased markedly as well. The median values of salivary arsenic in the 3 groups were 3.41, 11.20 and 22.91 μg/L, and the differences were significant (*P* < 0.05) (Table 1). The present results were similar to those of Yuan et al. [20] who analyzed the arsenic concentration in human saliva of 32 volunteers from Edmonton, Alberta, Canada, who had been exposed to background levels of arsenic less than 5 μg/L in drinking water. The mean value of the total arsenic in saliva was 0.79 μg/L. However, the saliva samples were collected from 301 residents of Ba Men, Inner Mongolia, China, who were exposed to arsenic concentrations up to 826 μg/L in drinking water, and the mean value of the total arsenic in these samples was 11.9 μg/L.

### Correlations among total arsenic concentrations in saliva, urine and drinking water

**Fig. 1:**
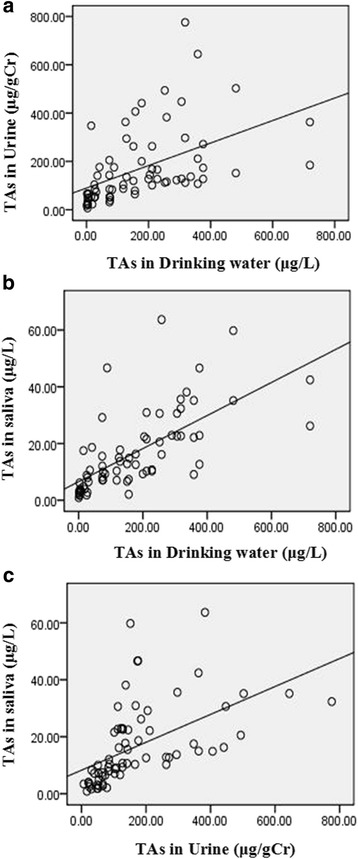
Relationship among total arsenic (TAs) in drinking water, urine and saliva. **a** Correlation of TAs between drinking water and urine (*r* = 0.686, *P* < 0.01, *n* = 70). **b** Correlation of TAs between saliva and drinking water (*r* = 0.674, *P* < 0.01, *n* = 70). **c** Correlation of TAs between urine and saliva (*r* = 0.794, *P* < 0.01, *n* = 70)

The present study compared the total arsenic concentrations in urine and drinking water by Spearman’s rank correlation analysis and revealed there was a significant positive association between them (*r* = 0.686, *P* < 0.05). Comparison of the total arsenic in saliva and drinking water with Pearson correlation analysis also showed that, similar to urinary arsenic, there was an obvious positive association between them, the correlation coefficient was 0.674, *P* < 0.05. A previous study has reported that there was a good correlation between the arsenic concentrations in drinking water and in saliva (*r* = 0.610), as well as between the arsenic concentration in drinking water and in urine (*r* = 0.644) [34]. Additionally, we compared the total arsenic in saliva and urine with Spearman’s rank correlation analysis and found that there was a significant positive correlation between them, the correlation coefficient was up to 0.794, *P* < 0.05 (Fig. 1). Our results were quite consisted with the report by Bhowmick et al., [35], who found that total arsenic concentration of saliva and urine also had a significant positive correlation by a case-control study in West Bengal, India. Their study also advocates that measurement of the forms of arsenic in saliva may additionally provide insight into the internal dose and any individual differences in susceptibility to arsenic exposure.

### Relationship between skin lesions and total arsenic in drinking water, urine and saliva

Arsenic tends to concentrate in ectodermal tissue such as the skin, hair and nails, and thus, skin lesions (both malignant and non-malignant lesions) were considered to be the most common adverse health effects associated with chronic arsenic exposure in humans [36]. In the present study, trained medical doctors conducted detailed physical examinations according to the Diagnosis Standards on Arsenicosis of China [25] to identify cases of different skin lesions. The results showed that there were 37 individuals with varying degrees of skin lesions among the 70 objects. We divided the crowd into two groups according to the presence or absence of skin lesions, and compared the total arsenic concentrations in drinking water, urine and saliva between the two groups by Student’s *t*-test. Table 2 showed the results of analysis indicating the concentrations of total arsenic in drinking water, urine and saliva in the group with skin lesions were significantly higher than those in the group with no skin lesions (*P* < 0.05). Before this study, a higher prevalence rate of arsenical skin lesions with a clear dose-response relationship was found among Bangladeshi populations ingesting arsenic contaminated water [37]. Additionally, Kile et al. [38] reported that there was a great risk of skin lesions associated with urinary arsenic. Our present results once again confirmed that there was an obvious correlation between skin lesions and arsenic present in drinking water and urine. It was worth mentioning that in the simultaneous analysis of the relationship between skin lesions and salivary arsenic, there was also a significant difference in salivary arsenic between the two groups, *P* < 0.05 (Table 2). Furthermore, there was an obvious positive association between salivary arsenic and total arsenic in drinking water and urine, which suggested that the total arsenic in saliva can be used as an effective biomarker of arsenic exposure.

**Table 2:** Relationship between skin lesions and total arsenic (TAs) concentrations in drinking water, urine and saliva

Skin lesions	Numbers	Arsenic in drinking water (μg/L)	Urinary arsenic (μg/gCr)	Salivary arsenic (μg/L)
Absent	33	111.99 ± 19.14	98.89 ± 14.52	9.21 ± 1.26
Present	37	225.97 ± 30.12^**^	233.69 ± 28.91^**^	23.18 ± 2.53^**^
*t* value	—	−3.194	−4.167	−4.938

** *p* < 0.01, compared with the skin lesions absent group, statistical analysis by Student’s *t*-test

### Arsenic species in urine and saliva of individuals

**Fig. 2:**
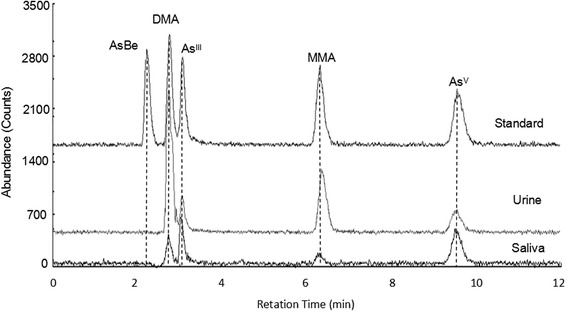
Chromatograms of arsenic species in the standard solution, urine sample and saliva sample obtained using HPLC-ICP-MS

We quantified the arsenic species in urine and saliva samples of individuals using HPLC-ICP/MS. As shown in Fig. 2, As^III^, As^V^, MMA, and DMA were detected in all of the urine samples, and in saliva samples, most of them contained detectable DMA and MMA, but the major species were iAs (As^III^ + As^V^). The median values of the four different species, sum As and total As levels between male and female were shown in Table 3. Comparison of urinary arsenic between male and female participants we can see that, even though the concentrations and distributions of As species in female were more higher than that of in male, there were no significant differences between them (*p* > 0.05), which was consisted with the study of Sun et al., [33]. However, Tseng et al., [39] detected the arsenic and its species in urine of 479 adults people (220 men and 259 women) found that women had a higher ability to methylate arsenic than men. The reason of these differences maybe because the sample individual numbers were fewer so we cannot exclude the possible contribution of gender differences in the study group. Besides, due to the demographic information of this study was limited, other influencing factors (e.g., age, BMI, living habits) of arsenic methylation capacity required to be further investigated.

**Table 3:** Concentrations of different arsenic species, Sum As and total arsenic (TAs) in urine and saliva samples

	Urine As (μg/gCr)		Saliva As (μg/L)	
	Median (95%CI)		Median (95%CI)	
	Male (37)	Female (33)	Male (37)	Female (33)
As^III^	14.3 (2.76–34.44)	16.60 (2.72–46.90)	10.08 (1.42–35.59)	8.78 (1.83–29.73)
As^V^	18.15 (4.53–55.58)	21.85 (4.28–54.74)	7.51 (1.78–19.00)	3.27 (ND^*^–15.05)
MMA	35.15 (6.24–101.22)	40.8 (9.4–167.76)	2.14 (0.70–3.34)	1.73 (ND–3.35)
DMA	85.75 (10.23–238.00)	103.60 (18.52–432.48)	3.03 (1.14–7.28)	1.83 (1.24–5.69)
Sum As^a^	153.45 (24.96–429.24)	198.45 (37.60–687.00)	28.23 (6.82–43.05)	22.14 (6.55–34.86)
TAs^b^	164.37 (27.05–447.24)	205.84 (48.25–775.73)	26.34 (3.32–63.67)	19.51 (1.90–35.61)

^a^Values are concentrations of sum of arsenic compounds detected in speciation analysis by HPLC-ICP-MS; ^b^ Values are total arsenic concentrations directly determined by ICP-MS; * ND, not detected (bellow LOD)

Similar to urinary arsenic, there was also no significant differences between male and female in salivary total arsenic and its species (As^III^, As^V^, MMA, and DMA) as shown in Table 3. Both in male and female, there was good agreement between SumAs and TAs which were directly detected by ICP-MS.

### Comparison of the proportion of arsenic species and methylation indices in urine and saliva

Even though some researchers had analyzed the total arsenic and arsenic species in human saliva, the comparison of arsenic species between urine and saliva has not yet been reported. Here, we analyzed the proportion of arsenic species and methylated indices in saliva and compared them with that of in urine for the first time. As shown in Table 4, the most excreted arsenic compound in urine was DMA, accounting for approximately 54.38% of the total, and the second excreted was MMA, about 23.14% of the total, there was only 21.65% of iAs (As^III^ + As^V^) species in urine samples. Which was consisted with the report by Hata et al., [40], who detected the arsenic and its species in urine of 165 married couples lived in the Pabna District in Bangladesh for more than 5 years, the iAs concentration in drinking water there was ranged 0.5-332 μgAs/L and the median value of arsenic species proportion in urine was DMA 73.2%, MMA 10.9%, iAs 15.9% respectively. However, in saliva samples, the proportion of arsenic species were obviously different with that of in urine samples. The most excreted arsenic compound in saliva was not DMA but iAs, the mean value of iAs proportion was 76.18%, DMA and MMA proportion were only 13.08 and 9.13%, respectively. Our results were similar to the study by Yuan et al., [20], who detected arsenic species for the first time in human saliva which was collected from 301 people lived in Ba Men, Inner Mongolia, China, where the drinking water also contained high levels of iAs. The mean percentages of individual arsenic species concentration over the mean total arsenic concentration were: iAs85%, MMA7%, and DMA4%. Subsequently, Lew et al., [23] analyzed arsenic species in saliva collected from 61 children who played on chromated copper arsenate (CCA) and non-CCA playgrounds also found that iAs was the most excreted arsenic compound.

**Table 4:** Proportion of different arsenic species (DMA, MMA, As^III^, and As^V^) and methylation indices in urine and saliva samples

	Proportion (%)				Methylation indices	
	DMA	MMA	As^III^	As^V^	PMI^a^	SMI^b^
Urine Samples						
Mean	54.38	23.14	8.98	12.67	0.78^**^	0.7^*^
Std. Deviation	1.02	0.32	0.40	0.69	0.01	0.01
Percentiles						
25	50.40	22.09	7.47	11.05	0.74	0.68
50	55.21	23.35	9.11	12.46	0.78	0.71
75	57.60	24.29	10.39	15.22	0.8	0.72
Saliva Samples						
Mean	13.08	9.13	48.22	27.97	0.22	0.55
Std. Deviation	1.41	1.12	5.43	3.17	0.02	0.05
Percentiles						
25	6.01	6.35	30.81	11.79	0.06	0.51
50	15.78	9.16	33.85	33.78	0.25	0.6
75	17.38	14.08	86.79	39.75	0.32	0.67

^a^Primary methylation index (PMI) calculated from (MMA + DMA)/total As (TAs), ^b^Secondary methylation index (SMI) calculated from DMA/(MMA + DMA) [33]

* *p* < 0.05, ** *p* < 0.01, compared with mean value in saliva samples by Student’s *t*-test

Besides, the primary methylation index (PMI) and second methylation index (SMI) in saliva were also significantly lower than that of in urine (Table 4). Suggested that most of the arsenic in saliva maybe not be metabolized and methylated. In our previous publications, we also found that saliva assay can be used as a useful tool for biological monitoring of total arsenic exposure, however, after consumption of organic arsenic (Chinese Seaweed), six species were detected in urine but only three species were detected in saliva samples, and most of them are unmethylated [41].

To further confirm the relationship of arsenic species and methylated indices between saliva and urine, we analyzed the correlation among them by Pearson’s correlation coefficient, unlike the obvious positive correlation of total arsenic in urine and saliva, no significant correlation were observed among the proportion of four species in urine and saliva (data not shown). As we all know that urine is considered as a surrogate for assessing arsenic metabolism in human body [42]. The low proportion of methylated arsenic species (MMA, DMA) in saliva and negative relationship with urine found in the present research suggested that although total arsenic level in human saliva can be used as a new biomarker of arsenic exposure, saliva may be not an efficient tool for understanding the metabolism of arsenic in the body. Hines et al., [43] analyzed serum, saliva, urine, and milk for the oxidative phthalate metabolites of 33 lactating North Carolina women also found that phthalate metabolites are most frequently detected in urine, but unlikely to be detectable in milk and saliva.

### Benefits of saliva as a biomonitoring tool for assessing total arsenic exposure

The use of saliva as a biomonitoring tool for assessing total arsenic exposure has several benefits compared with other current biomonitoring tools. For example, researchers often can not directly collect urine samples due to personal privacy issues, therefore, it is difficult to ensure the authenticity of samples [44]. In contrast, saliva samples can be easily collected, and particularly advantageous when children and menstruating women are involved. Blood may be the best tissue for monitoring most pollutants, but it is not suitable for arsenic monitoring because the half-life of arsenic and its compounds in blood is very short as most eliminated from the blood in a few hours [45]. In addition, the composition of blood is quite complex and its collection is invasive. However, the arsenic in saliva is relatively stable and the collection method for saliva is simple and non-invasive [41], which makes it more suitable for population studies. Hair and nail samples are easy to collect, but they are vulnerable to contamination by external environmental pollutants, so it is difficult to accurately determine the exposure dose for arsenic in the body [46]. Compared with hair and nail samples, saliva samples are less affected by external interference.

### Limitation of saliva as a biomonitoring tool for assessing arsnenic metabolites in human body

Different with the strong positive correlation of total arsenic in saliva and urine, for arsenic metabolites, obviously different distribution of the four main arsenic species (As^III^, As^V^, MMA, and DMA) between saliva and urine was found in the present study. For urinary arsenic, the most excreted compounds were methylated arsenic (DMA and MMA), only about 10% ~ 20% of iAs was existed both in our data and other reports [33, 39, 40]. However, in the same individual, only about 20% of methylated compounds (DMA and MMA) were detected in saliva samples, and some of them were not detected, conversely, iAs compound percentage was as high as 76%. Besides, arsenic level in saliva was also considered to be lower and small variation [24] which renders further identifying the kinds of its species difficult. However, in another study reported by Bhowmick et al., [47] showed that, the dominant fraction of As in saliva consists of inorganic As but, interestingly, significant associations were observed between the total daily As intake and the concentrations of methylated species in the saliva samples. Thus, whether or not saliva can be used as an efficient tool for assessing arsenic metabolites in humans exposed to arsenic requires further investigation.

## Conclusions

In summary, the present study has shown that total arsenic concentration in saliva exists a strong positive correlation with total urinary arsenic, drinking water arsenic and different skin lesions. Furthermore, the collection method for saliva is simple and non-invasive, which makes it can be used as a new biomarker for monitoring total arsenic exposure in the crowd. For arsenic speciation analysis, different with urinary arsenic species, most arsenic species in saliva are not methylated. The PMI, SMI and proportion of the four different species (As^III^, As^V^, MMA, and DMA) in saliva showed a poor relationship with that of in urine. In addition, the concentration of arsenic in saliva are considerably lower than in urine, which renders further identifying the kinds of its metabolites difficult. Thus, saliva assay is probably a useful tool for biological monitoring of total arsenic exposure in the crowd rather than an efficient tool for assessing arsenic metabolites in humans exposed to arsenic.

## Abbreviations

ANOVA: One-way analysis of variance
AsBe: Arsenobetaine
AsIII: Sodium arsenite
AsV: Sodium arsenate
CCA: Chromated copper arsenate
Cr: Creatinine
DMA: Dimethylarsinate
HPLC-ICP/MS: High-performance liquid chromatography-inductively coupled plasma-mass spectrometry
IARC: International agency for research on cancer
LOD: Limit of detection
LOQ: Limit of quantitation
MMA: Methylarsonic acid
PMI: Primary methylation index
SMI: Second methylation index
